# Variation in Disease Susceptibility Is Associated With Immunogenetics in a Threatened Amphibian

**DOI:** 10.1111/eva.70348

**Published:** 2026-10-28

**Authors:** Rebecca A. Clemons, Anat M. Belasen, Bennett M. Hardy, Erin Muths, Larissa L. Bailey, W. Chris Funk, Kelly R. Zamudio

**Affiliations:** ^1^ Department of Integrative Biology The University of Texas at Austin Austin Texas USA; ^2^ School of Biological Sciences The University of Oklahoma Norman Oklahoma USA; ^3^ San Diego Natural History Museum San Diego California USA; ^4^ U.S. Geological Survey Fort Collins Colorado USA; ^5^ Department of Fish, Wildlife, and Conservation Biology Colorado State University Fort Collins Colorado USA; ^6^ Graduate Degree Program in Ecology Colorado State University Fort Collins Colorado USA; ^7^ Department of Biology Colorado State University Fort Collins Colorado USA

**Keywords:** boreal toad, chytridiomycosis, conservation, major histocompatibility complex, MHC

## Abstract

Emerging infectious diseases affect wildlife populations around the world, making effective conservation strategies essential to the long‐term persistence of threatened species. Understanding the drivers of protective host responses to pathogens can help guide conservation efforts. Species that vary intraspecifically in their susceptibility to pathogens are often best for identifying significant drivers, as this reduces potentially confounding effects of highly divergent genetic and adaptive backgrounds. Boreal toads ( *Anaxyrus boreas boreas* ) exhibit such intraspecific variation in disease susceptibility. This species ranges across western North America and declines due to the fungal pathogen *Batrachochytrium dendrobatidis* (Bd) have varied in severity across populations. Previous experimental work shows that some populations of boreal toads are better able to tolerate Bd than others, though the mechanisms are currently unclear. We sequenced the immune locus MHC IIβ exon 2 across tolerant and susceptible populations of boreal toads to determine if host genetics is correlated with Bd susceptibility in this system. We found that tolerant populations differ from susceptible populations at this locus. Specifically, tolerant populations had several distinct MHC alleles, higher observed heterozygosity, and a higher proportion of alleles belonging to one of two functional supertypes. Using these data, we predicted the Bd response of additional boreal toad populations with unknown susceptibility. Boreal toad conservation efforts will benefit from the ability to identify at‐risk populations and individuals with genetic signatures of Bd tolerance for use in captive breeding and reintroduction efforts.

## 1 Introduction

Wildlife populations around the globe are threatened by emerging infectious diseases (Daszak et al. 2000; Smith et al. 2006, 2009). Anthropogenic stressors, including climate change and habitat loss, can exacerbate disease‐related declines (Martin et al. 2010), making effective conservation management strategies a priority. In light of these declines, it is crucial to understand what factors drive host responses to pathogens and whether that response is sufficient for host persistence. Many factors can influence a host's response to a pathogen, including but not limited to environmental conditions (Savage et al. 2011; Clare et al. 2016), community composition (Venesky et al. 2014), anthropogenic stressors (Rohr et al. 2013), and the host's immune capabilities (Grogan, Robert, et al. 2018). Determining which factors are most influential in each wildlife‐disease system can aid in determining the most efficient use of limited conservation resources.

Wildlife hosts can vary in their response to pathogens both interspecifically and intraspecifically. Millions of years of evolution can separate even closely related species, obscuring potential differences in genetic background, exposure history, and adaptation that can underlie differences in infection outcomes. This challenges our ability to pinpoint factors that drive variation among species. Because of these underlying differences, investigating intraspecific variation in host persistence is often the most informative for revealing mechanisms of host resistance or tolerance. Additionally, species with intraspecific variation are good targets for conservation management as individuals can be moved among populations to facilitate potential genetic rescue. Intraspecific variation in susceptibility to pathogens has been documented and explored in numerous systems (Savage et al. 2011; Bonneaud et al. 2019; Knutie 2018; Rocke et al. 2012); in some cases, environmental factors play a primary role in determining disease outcomes (Kriger et al. 2007; Murdock et al. 2013), while in other cases, the outcomes seem to be primarily driven by host immune response (Grogan, Cashins, et al. 2018; Savage and Zamudio 2016). Disentangling these drivers can provide insight into the mechanisms hosts use for pathogen resistance (i.e., limiting pathogen load) or tolerance (i.e., limiting pathogen‐induced damage) (Råberg et al. 2008), paving the way to the development of specific conservation interventions that support host persistence.

Populations of amphibians worldwide have suffered severe declines and, in some cases, extinction due to the emerging infectious disease chytridiomycosis, caused by the fungal pathogen *Batrachochytrium dendrobatidis* (Bd) (Scheele et al. 2019). While some species and populations are extremely susceptible to Bd, others exhibit resistance or tolerance to infection and are able to persist despite the presence of Bd (Pilliod et al. 2010; Knapp et al. 2024). Currently, the mechanisms that drive these differences are unclear in many systems (but see Belasen et al. (2024) and Savage and Zamudio (2016)). The identification of these drivers, particularly in species with intraspecific variation, could provide a powerful tool for predicting disease response in populations where the impacts of disease are unknown. One species that exhibits intraspecific variation in Bd susceptibility is the boreal toad ( *Anaxyrus boreas boreas* ); populations in the southeastern part of its range have experienced dramatic declines while other populations persist with Bd (Pilliod et al. 2010; Hossack et al. 2020; Mosher et al. 2018), providing an excellent study system for exploring drivers of disease susceptibility. If disease response in this system is primarily driven by environmental factors, climate variables can help identify at‐risk populations and sites for potential reintroductions. If disease response is driven primarily by host immune response, genetic screening could identify at‐risk populations and good source populations for captive breeding and reintroduction efforts.

Variation in host immune response can play a large role in determining disease outcomes, especially in cases where populations experience similar environmental conditions. However, directly measuring immune response is challenging as the immune system consists of many interacting components that function differently during various stages of an infection. Additionally, controlled experimental exposures can be logistically and/or ethically impractical. Therefore, sequencing host DNA to look for variation in immune genes can be used as a proxy for measuring variation in immune function (Savage and Zamudio 2011). One group of genes that is ideal for this type of analysis is the Major Histocompatibility Complex (MHC). MHC is involved in the presentation of pathogen‐derived molecules (antigens) to immune cells which go on to activate or amplify an adaptive immune response (Bevan 1987). MHC class I typically targets intracellular pathogens (e.g., viruses) while MHC class II targets extracellular pathogens (e.g., fungi) (Bevan 1987). MHC loci exhibit a large degree of variation within and between individuals to encompass the wide array of pathogens a host might encounter in its lifetime (Savage and Zamudio 2011). Within a population, having an optimal diversity of MHC alleles can be advantageous, as this allows for a response to a wide array of pathogens while minimizing the risks of auto‐immune responses due to having too many MHC alleles (Oliver and Piertney 2012; Kalbe et al. 2008). Additionally, having specific MHC alleles may also be advantageous if these alleles are effective against particular pathogens that hosts might encounter frequently (Savage and Zamudio 2011). MHC diversity can also be mediated by demography, gene flow, and other evolutionary processes. These processes can contribute to underlying variation in MHC diversity among populations, which then experience selection by or responses to emerging disease differently.

Variation at MHC loci has been shown to be important for numerous species of amphibians facing threats from disease (Savage and Zamudio 2011; Bataille et al. 2015; Fu et al. 2023; Belasen et al. 2022). Amphibians in Brazil's Atlantic Forest with heterozygous MHC II loci had lower Bd prevalence compared to MHC homozygotes (Belasen et al. 2022). Similarly, *Rana yavapaiensis* individuals with heterozygous MHC II loci had higher rates of survival following Bd infections (Savage and Zamudio 2011). This study also revealed the presence of certain protective MHC alleles in *R. yavapaiensis* that were associated with survival following Bd infection (Savage and Zamudio 2011). MHC screening has been used to try to predict disease susceptibility in humans, where certain MHC genotypes have been associated with the severity of various diseases (Matzaraki et al. 2017; Davies et al. 2012). These findings have led to an interest in using MHC to predict disease susceptibility in amphibians.

In this study, we sequence an MHC II locus across populations of boreal toads ( *Anaxyrus boreas boreas* ) to test if MHC diversity or allele distribution is associated with Bd susceptibility. A previous Bd exposure experiment has shown that populations continue to exhibit differences in susceptibility in the laboratory, indicating that host immune response is contributing to variation in susceptibility rather than just the environment (Hardy et al. 2025). We hypothesize that individuals from populations that have shown similar responses to Bd (tolerant or susceptible) will have similar MHC profiles (i.e., allele distribution and heterozygosity), and that MHC profiles will differ between tolerant and susceptible populations. Based on findings from other amphibian systems (Savage and Zamudio 2011; Belasen et al. 2022), we hypothesize that tolerant populations will have higher heterozygosity and have unique alleles that are absent from susceptible populations. Finally, we use MHC profiles to predict whether populations with unknown Bd responses are likely to be tolerant or susceptible.

## 2 Methods

### 2.1 Study System

The boreal toad, *Anaxyrus boreas boreas* , ranges across western North America and has declined due to Bd in part of its range (Mosher et al. 2018; Muths et al. 2008). Boreal toads have been listed as a Species of Greatest Conservation Need Tier I in Colorado, Wyoming, and New Mexico (Wellman et al. 2024). This species shows population‐level variation in persistence following Bd exposure as many southern populations have experienced declines (Mosher et al. 2018; Scherer et al. 2005; Hardy et al. 2023) while northern and western populations persist (Pilliod et al. 2010; Hossack et al. 2020, 2015). Hardy et al. (2025) found that populations of boreal toads in western Wyoming had greater tolerance to Bd than Colorado populations and were more likely to survive their infections at equivalent Bd loads in laboratory trials. Further, the authors found mixed evidence for Bd resistance among Wyoming populations: individuals from Wyoming and Colorado populations had no difference in peak Bd loads, although Wyoming populations reached these peak loads more slowly (Hardy et al. 2025). These populations exhibited variation in susceptibility in a controlled laboratory setting, indicating that variation is largely being driven by host immune function rather than solely an external factor, like the environment.

We included individuals from the same four populations experimentally tested in Hardy et al. (2025) and categorized them as either Bd tolerant or Bd susceptible according to those experimental results. We chose to categorize Wyoming populations as tolerant rather than resistant due to the stronger evidence for Bd tolerance in Hardy et al. (2025). We included an additional seven populations from Montana, Colorado, Idaho, and Utah that in most cases have been exposed to Bd and in some cases show reductions in population size, but their Bd susceptibility has not been measured directly (Figure 1, Table S1).

**FIGURE 1:**
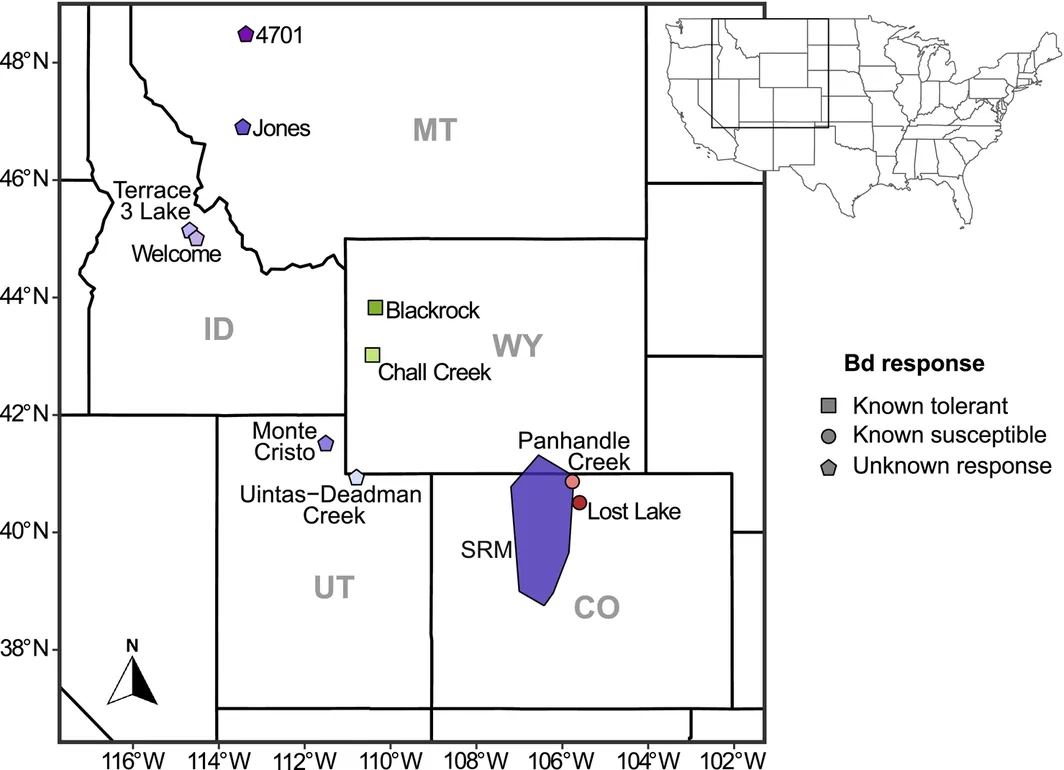
Map of boreal toad ( *Anaxyrus boreas boreas* ) population locations. The polygon represents the localities of individuals included in the SRM “population”, which is a population with unknown response. CO, Colorado; ID, Idaho; MT, Montana; UT, Utah; WY, Wyoming.

### 2.2 Experimentally Tested Populations (*n* = 4, Hardy et al. 2025)

The Wyoming populations, Blackrock‐WY (*n* = 14) and Chall Creek‐WY (*n* = 14), are categorized as Bd tolerant based on experimental exposure (Hardy et al. 2025). In this case, tolerance indicates that individuals from these populations are able to better survive high Bd loads compared to individuals from susceptible populations, but can still be infected by Bd and succumb to severe infections. Bd negatively affects the survival of individuals in the Blackrock‐WY population; the number of individuals in that population declined by 5%–7% per year during a 6 year study (Pilliod et al. 2010; Muths et al. 2020). However, this gradual decline is far less dramatic than declines that have been documented in Colorado populations (Mosher et al. 2018; Scherer et al. 2005; Hardy et al. 2023), supporting experimental results of higher tolerance in WY populations (Hardy et al. 2025). Similarly, Bd is prevalent (~70%) in the Chall Creek‐WY population, but there is little impact on annual survival probabilities (Barrile et al. 2021). Two of the Colorado populations, Lost Lake‐CO (*n* = 16) and Panhandle Creek‐CO (*n* = 12), are categorized as Bd susceptible based on experimental exposure (Hardy et al. 2025). Bd was first detected in the Lost Lake‐CO population in 1998, and dramatic declines in survival probabilities and abundance were observed immediately after (Scherer et al. 2005; Muths et al. 2003). This population has never rebounded. Bd has also been detected in the Panhandle Creek‐CO population, but no demographic monitoring has been conducted at this location (Hardy et al. 2025).

### 2.3 Additional Populations (*n* = 7, Not Tested Experimentally)

Samples from additional (*n* = 15) extant southern Rocky Mountain populations (i.e., not including Lost Lake‐CO or Panhandle Creek‐CO) were combined into the SRM “population”, and are were also used in analyses reported in Trumbo et al. (2023). We combined these individuals into a single southern Rocky Mountain “population” (*n* = 27) due to low sample size (i.e., few individuals in each population) (Table S2) and minimal genetic structure among these populations in Colorado and southern Wyoming according to neutral markers (Trumbo et al. 2023). Immune markers such as MHC may show more distinct population structure due to local pathogen‐mediated selection (Ekblom et al. 2007). Bd has been detected in these populations since approximately 2000 (Trumbo et al. 2023), and several of the populations have experienced dramatic declines similar to those reported for Lost Lake‐CO (Hardy et al. 2023). However, experimental exposures have been limited (Carey et al. 2006), so we conservatively suggest the specific response of this “population” to Bd is unknown.

The Montana populations, Jones‐MT (*n* = 16) and 4701‐MT (*n* = 15), have been monitored for Bd since 2004 and 2008, respectively (Hossack et al. 2020). Both of these populations have experienced declines, and while Bd has been shown to negatively affect survival, population decline has not been linked directly to Bd prevalence (Hossack et al. 2020; Kain et al. 2025). The Idaho populations, Terrace 3 Lake‐ID (*n* = 4) and Welcome‐ID (*n* = 13), have no published records of Bd occurrence, but both populations are small and at risk of extirpation from stochastic events (e.g., drought, but could include the effects of disease) (D. Pilliod, personal communication). The Utah populations, Monte Cristo‐UT (*n* = 5) and Uintas‐Deadman Creek‐UT (*n* = 7), have been monitored periodically since the early 2000s. Bd has been documented in the state since 2001 (Thompson et al. 2004) and locally in the Uinta Mountains since 2006 (P. Thompson, personal communication). No published demographic information is available for either population.

### 2.4 Sample Collection

Buccal swabs and tissues (i.e., toe and tail clips from adults and tadpoles, respectively) from the SRM “population”, were collected in Colorado and southern Wyoming in 2018–2020 by state and federal biologists (Trumbo et al. 2023). Buccal swabs, tadpole tail clips, and adult toe clips were collected from Blackrock‐WY, Lost Lake‐CO, Panhandle Creek‐CO, 4701‐MT, Jones‐MT, Monte Cristo‐UT, and Uintas‐Deadman Creek‐UT in 2018; from Terrace 3 Lake‐ID and Welcome‐ID in 2019; and from Chall Creek‐WY in 2023. We collected samples across all life stages to maximize sample sizes and because we are investigating population level susceptibility. All samples were stored in ethanol at −20°C until DNA extraction.

### 2.5 Genome Walking and Primer Design

We used the DNeasy Blood and Tissue Kit (Qiagen) to extract DNA from toe clips, tadpole tail clips, and buccal swabs. We used a genome walking approach (Shapter and Waters 2014) to design primers to target the second exon of the boreal toad MHC class II β chain. Genome walking allows us to use existing primers that target a small portion of our locus of interest and “walk out” to capture unknown flanking regions for more specific primer design. We chose this particular locus for sequencing because MHC class II is involved in responses to extracellular fungal pathogens like Bd (Bevan 1987). Exon two of the β chain is highly variable, makes up a portion of the MHC antigen‐binding region, and has been shown to be specifically informative for Bd response in other amphibian species (Savage and Zamudio 2011; Bataille et al. 2015; Belasen et al. 2022, 2019).

We performed genome walking with the GenomeWalker 2.0 kit (Takara Bio Inc.) using HaeIII and SspI restriction enzymes (New England Biolabs) for the initial digestion of our samples, as the restriction enzymes included in the Takara kit did not yield successful products. We used BCF6 and BobomSR (May and Beebee 2009) as the gene‐specific primers, as these primers amplified an approximately 100 bp region of the boreal toad MHC IIβ exon 2. Using the results generated from genome walking followed by cloning and Sanger sequencing, we designed primers to target a single copy of the boreal toad MHC IIβ exon 2. We identified the intron/exon boundaries by aligning our genome walking results to a reference *Rhinella marina* MHC IIβ exon 2 sequence. We ensured that no stop codons were present between these points and designed primers that overlapped with these boundaries. By including part of the flanking introns in our primer design, our primers should only amplify a single copy of this exon and should not amplify other MHC paralogs that may be present in the genome. In total, our primers target a 331 bp region (Anbobo_F: GTGTAGTGTGAGCTCCGTGT and Anbobo_R: GCCTCACCTTTTCTTTCTATAGC).

### 2.6 Library Preparation and Sequencing

We performed PCR using ExTaq polymerase (New England Biolabs) and our boundary primers on all 154 boreal toad samples. The PCR conditions were 95°C for 3 min, 40 cycles of (95°C for 30 s, 57°C for 30 s, 72°C for 1 min), and 72°C for 5 min. A subset of samples that failed with initial PCR attempts was amplified using a modified PCR protocol with a 47°C annealing temperature. We cleaned our PCR products using a 1.8X volume of AMPure XP beads (Beckman Coulter Inc.) before library preparation.

We used the Watchmaker DNA Library Preparation Kit (Watchmaker Genomics) to prepare our PCR amplicons for sequencing. We used xGen Stubby Adapters and UDI primers (Integrated DNA Technologies Inc.) to index our samples, then visually checked libraries using electrophoresis on a 1% agarose gel. A subset of samples had an additional smaller band (approximately 250 bp); for these samples, we performed an additional cleanup using SPRIselect beads (Beckman Coulter Inc.) at 0.6X volume. All samples were pooled in equimolar volumes and sequenced on the NextSeq1000 platform with 300 bp paired‐end reads at The University of Texas at Austin Genomic Sequencing and Analysis Facility.

### 2.7 MHC Allele Assignment

We used the Mothur MiSeq pipeline to process our reads and identify unique MHC alleles using a 99% OTU similarity cutoff (Schloss et al. 2009). We opted to use the Mothur pipeline as opposed to an MHC‐specific pipeline (such as AmpliSAS) because it is customizable and repeatable. Mothur performs the same basic functions as these MHC‐specific pipelines: clustering reads into OTUs (alleles) while minimizing the impacts of sequencing error. In most cases, many OTUs (alleles) were recovered in each individual, but the majority of these were likely due to sequencing or PCR errors as they showed extremely low sequence coverage (number of sequence reads). We assigned the most likely alleles within individuals based on the number of reads, and in the majority of cases one or two alleles had significantly higher read depth than others that appeared in the sample (1–3 orders of magnitude higher) (Table S3). We opted to use an order of magnitude cutoff to genotype each sample due to the large variation in read depth between samples and our a priori knowledge that each sample should only contain one or two unique alleles. Alleles were considered true alleles if they appeared with significant read depth in at least two individuals or comprised at least 40% of reads within one individual.

For individuals that appeared putatively heterozygous based on read depth, we calculated the ratio between the allele with the highest coverage to the allele with the next highest coverage, similar to the approach used by the Stacks genotypes program (https://catchenlab.life.illinois.edu/stacks/comp/genotypes.php). In four individuals, the number of reads for the allele with the second highest coverage fell outside of 2 standard deviations of the mean (≥ 3.53X difference); this second allele was thus dropped, and these individuals were considered homozygous for the allele with the highest coverage. We removed 11 individuals from the final dataset for which allele assignment could not be confidently performed. These individuals appeared to have 3 or more alleles with equivalent read depth, potentially due to non‐specific primer binding to MHC paralogs or sequencing error. Most of these individuals had overall lower sequence coverage compared to individuals where confident allele assignment was possible.

### 2.8 Evaluation of MHC Diversity and Selection

To visualize the distribution of alleles across the populations, we created a haplotype network using the pegas package in R v4.3.2 (Paradis 2010; R Core Team 2021). We also used pegas to calculate summary statistics for each population including observed heterozygosity, expected heterozygosity, allelic richness, Tajima's D, and nucleotide diversity (*π*). We used the heirfstat package in R to calculate pairwise MHC fixation index (*F*_ST_) values for each of the populations (Goudet et al. 2022). We used the vegan package in R to perform a mantel test to determine if *F*_ST_ comparisons between known‐tolerant and known‐susceptible populations were correlated with geographic distance between populations (Oksanen 2025). To estimate if allele frequencies were significantly different between known‐tolerant and known‐susceptible populations, we used a Fisher's exact test with a simulated *p*‐value using 2000 replicates. Due to large variation in sample size, we did not include any of the unknown populations in statistical tests.

We used Geneious Prime v2024.0.7 (https://www.geneious.com) to translate alleles into amino acid sequences and visualize polymorphism in the MHC alignment. We used a fixed effects likelihood (FEL) model implemented on the Datamonkey webserver to estimate sites in the amino acid alignment that are under selection (Kosakovsky Pond and Frost 2005a, 2005b). We then aligned the amino acid sequences to a subset of additional anuran species included in Bataille et al. (2015) to identify the location of putative antigen‐binding sites (i.e., sites that are directly in contact with antigens and thus directly impact pathogen response).

Using the antigen‐binding sites identified from the alignment, we clustered the boreal toad MHC alleles into supertypes (i.e., groups of alleles with functional similarity in MHC binding specificity) to evaluate immunogenetic functional diversity. For each variable site (9 out of 13 total), we categorized the physiochemical properties of the amino acid present by assigning five z‐descriptor values (Sandberg et al. 1998). For one site (site 64), we assigned z‐descriptor values of zero to any allele that had a gap at this position. We also ran an additional analysis excluding this site entirely. We then used a discriminant analysis of principal components (DAPC) implemented in the adegenet package in R to cluster alleles into distinct supertypes using the z‐descriptor values (Jombart et al. 2010). We used Fisher's exact tests to determine if observed heterozygosity and supertype proportions were significantly different between known‐tolerant and known‐susceptible populations. To control for multiple comparisons, we performed a Benjamini‐Hochberg correction and reported the adjusted *p*‐values for all statistical tests (Benjamini and Hochberg 1995).

## 3 Results

### 3.1 MHC Allele Distribution

We recovered 16 unique MHC alleles from 143 individuals across 11 populations (Figure 2). Sample size per population ranged from *n* = 4 (Terrace 3 Lake‐ID) to *n* = 27 (SRM) individuals (Table S1). The populations were split into three categories based on Hardy et al. (2025): known Bd tolerant (*n* = 2), known Bd susceptible (*n* = 2), and unknown response to Bd (*n* = 7). Based on the distribution of alleles across the populations, we categorized several alleles as putative tolerance alleles (4, 5, 8, 9, and 13) because they primarily occurred in known‐tolerant populations and populations with unknown response. Individuals from the known‐susceptible populations had only the most common alleles across our dataset (1, 2, 3, 4, and 6). Allele frequencies were significantly different between known‐tolerant and known‐susceptible populations (*p* = 0.001). The remaining alleles (7, 10, 11, 12, 14, 15, and 16) occurred relatively rarely only in populations with unknown response (Figure 2).

**FIGURE 2:**
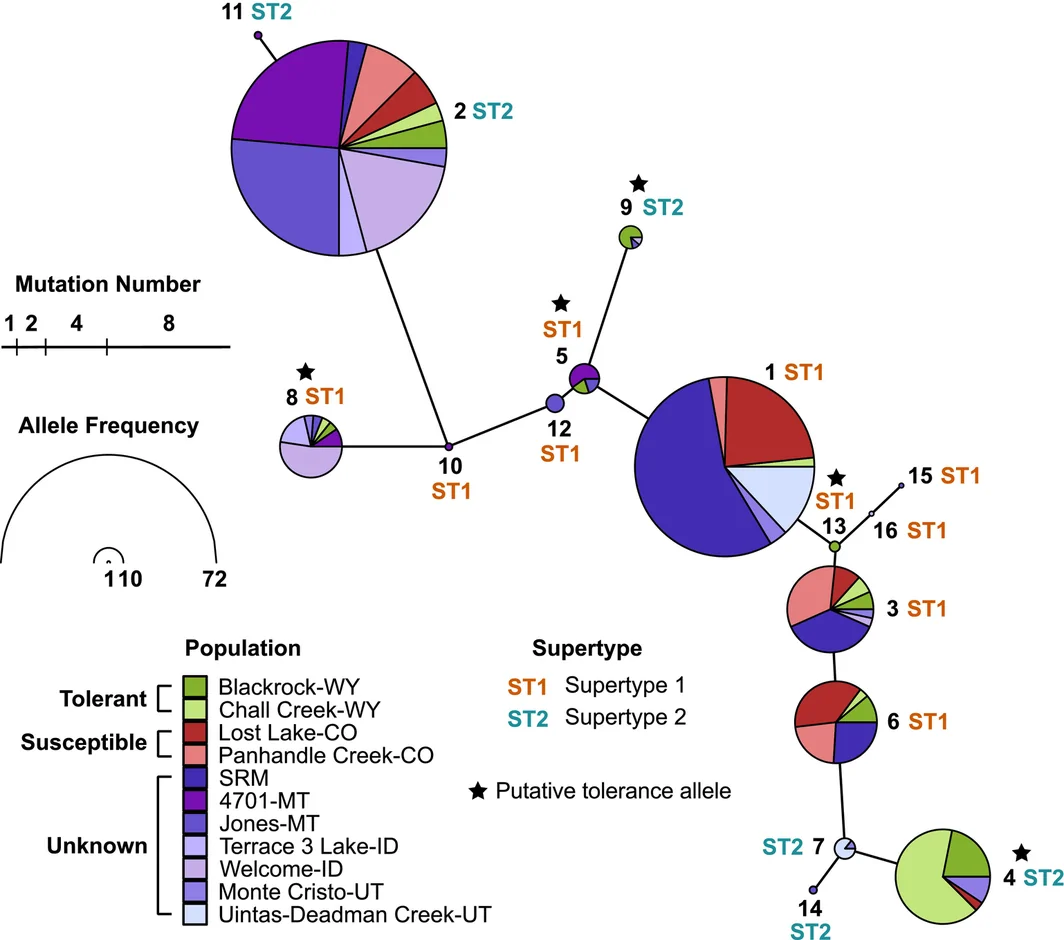
Haplotype network showing the distribution of alleles across populations. Each circle represents a unique allele: the black number is the allele number and the size of the circle represents the allele frequency (the number of times that allele appears across the dataset). Colors within the circles represent the populations where each allele is present, with the size of the slice representing the abundance of that allele in that population. The length of the branches between circles represents the mutation number (how many mutations separate alleles), with shorter branches indicating fewer mutations and therefore greater genetic similarity. See combined legend for scales for circle size/allele frequency and branch length/mutation number. The supertype of each allele is indicated next to the allele number. Putative tolerance alleles are indicated with a star.

### 3.2 MHC Diversity

Observed heterozygosity was 6.67× higher (*p* = 0.01, 95% CI: 1.67–33.49) in the known‐tolerant populations (42%–64%) compared to known‐susceptible populations (0%–25%). Populations with unknown response had a wide range of observed heterozygosity (13%–100%) (Figure 3). Putative tolerance alleles were present in five populations with unknown response (Monte Cristo‐UT, Terrace 3 Lake‐ID, Welcome‐ID, Jones‐MT, and 4701‐MT) and absent in the remaining two populations with unknown response (Uintas‐Deadman Creek‐UT and SRM).

**FIGURE 3:**
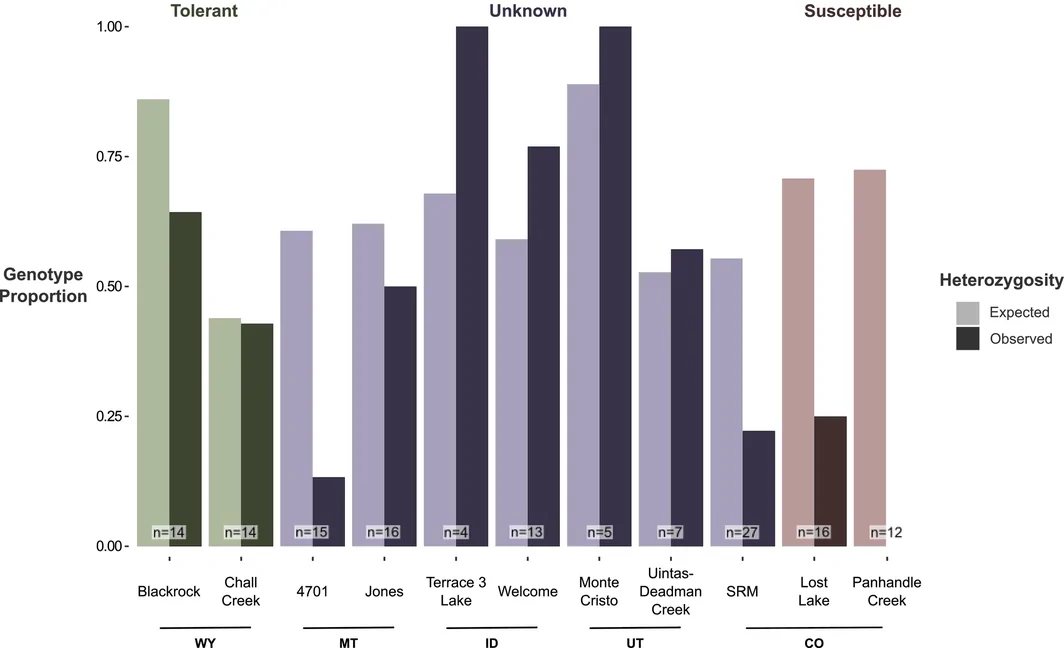
Observed and expected heterozygosity of each population. Lighter bars represent the expected heterozygosity of each population, while darker bars represent the observed heterozygosity. Numbers within bars represent the number of individuals per population. Panhandle Creek‐CO has an observed heterozygosity of zero. Observed heterozygosity is significantly higher in known‐tolerant populations compared to known‐susceptible populations (*p* = 0.001).

Tajima's D values were not significantly different from 0 and showed no evidence of bottlenecks in any of the populations at this locus (Table S1). Expected heterozygosity was similar between known‐tolerant (0.44–0.86), known‐susceptible (0.71–0.72), and populations with unknown response (0.53–0.89) (Figure 3, Table S1). Nucleotide diversity (*π*) was also similar among known‐tolerant (0.025–0.045), known‐susceptible (0.029–0.044), and populations with unknown response (0.014–0.054). Raw allelic richness was slightly higher in known‐tolerant populations (6–8 alleles) compared to known‐susceptible (4–5 alleles) and populations with unknown response (2–7 alleles), but was not significantly different when corrected for sample size (Table S1). *F*_ST_ was highest when comparing tolerant to susceptible populations (0.25 ± 0.06 SE). In contrast, *F*_ST_ was lower between the two tolerant populations (0.17) and between the two susceptible populations (0.09) (Table S4). *F*_ST_ was not significantly correlated with geographic distance when comparing known‐tolerant and known‐susceptible populations (*p* = 0.4, *r* = 0.143).

### 3.3 Selection on MHC and Supertyping

The FEL analyses indicated that four sites in the amino acid alignment were under diversifying positive selection. These were sites 23 (*p* = 0.03), 53 (*p* = 0.03), 60 (*p* = 0.03), and 64 (*p* = 0.03). All four of these sites aligned with putative antigen‐binding sites (Bataille et al. 2015) (Figure 4).

**FIGURE 4:**
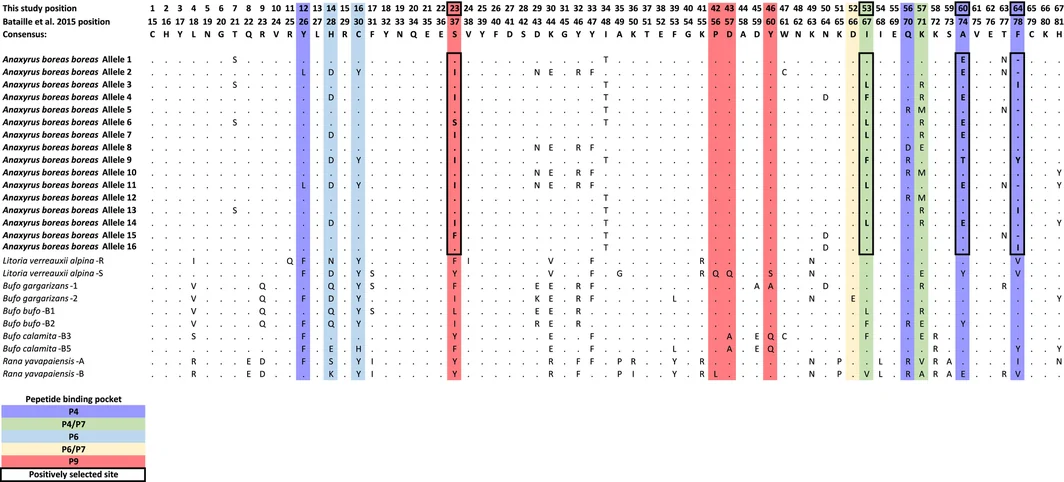
Alignment of boreal toad amino acid sequences with other species from Bataille et al. (2015). Dots represent agreement with the consensus sequence and dashes represent gaps in the alignment. Colored sites represent putative antigen‐binding sites with colors representing their respective peptide binding pockets. Bolded sites (23, 53, 60, 64) are sites under diversifying positive selection based on a fixed effects likelihood (FEL) analysis of the boreal toad ( *Anaxyrus boreas boreas* ) sequence alignment.

Based on nine variable antigen‐binding sites, the 16 alleles clustered into two distinct supertypes, which we name supertype one and supertype two to differentiate between them. The removal of site 64, which contained gaps in several alleles, resulted in the same clustering pattern. Alleles belonging to supertype two were 9.97× more common (*p* < 0.001, 95% CI: 3.93–27.2) in individuals from known‐tolerant populations (60%–82% of alleles) relative to known‐susceptible populations (15%–25% of alleles). Supertype two alleles ranged from 3% to 68% of alleles in populations with unknown response (Figure 5).

**FIGURE 5:**
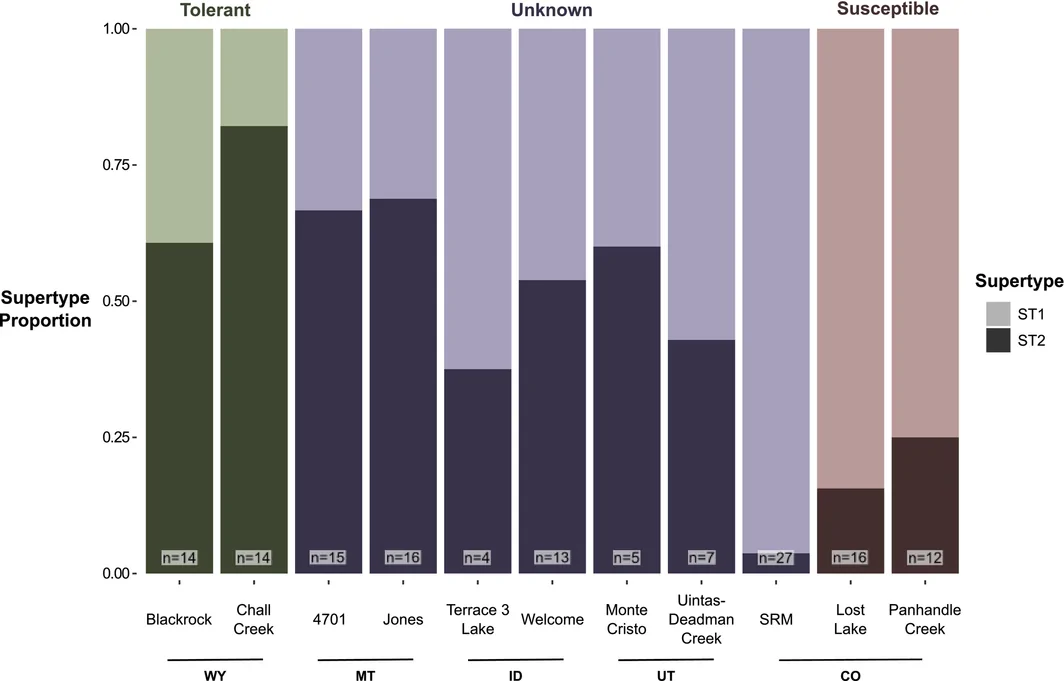
Proportion of supertypes in each population. The lighter portion of the bar represents the proportion of alleles belonging to supertype one, while the darker portion of the bar represents the proportion of alleles belonging to supertype two. Numbers within bars represent the number of individuals per population. The proportion of supertype 2 alleles is significantly higher in known‐tolerant populations compared to known‐susceptible populations (*p* < 0.001).

## 4 Discussion

We sequenced an immune locus, MHC IIβ exon 2, across populations of the boreal toad ( *Anaxyrus boreas boreas* ) to investigate if MHC variation is associated with disease susceptibility in this system. We recovered 16 unique MHC alleles across the 11 target populations (Figure 2). We found that MHC differed significantly between the two known‐tolerant and two known‐susceptible populations described in Hardy et al. (2025). We then compared the MHC profiles of these known populations to several additional populations to try to predict their Bd response and identify populations of conservation concern.

### 4.1 Identification of Putative Tolerance Alleles

Based on the distribution of alleles across populations, we have identified several alleles that may increase tolerance to Bd. These alleles are present in known‐tolerant populations and almost entirely absent in known‐susceptible populations. The presence of these alleles in populations with unknown responses may signal Bd tolerance (Figure 2). These alleles may also be contributing to Bd resistance, although we refer to them here as putative tolerance alleles due to the stronger evidence for Bd tolerance rather than Bd resistance among Wyoming populations in Hardy et al. (2025). Unfortunately, we do not have Bd infection data for the individuals genotyped in this study, so we are unable to estimate relative risk of Bd infection for particular alleles. We designated allele four as a putative tolerance allele despite the fact that it appears in a single known‐susceptible population (one copy, in one individual from Lost Lake‐CO; 3% of the total abundance of allele four). In contrast, 88% of the abundance of allele four is from known‐tolerant populations, and 9% from a population with unknown Bd response (Figure 2). Five of the seven populations with unknown responses had at least one putative tolerance allele, indicating that these populations may be better protected against Bd compared to known‐susceptible populations.

If these alleles are facilitating tolerance to Bd, a deadly pathogen that negatively impacts host fitness, we may expect them to be fixed in tolerant populations. However, we do not see fixation of MHC alleles in any of the populations. We hypothesize that these putative tolerance alleles are not fixed due to other forces acting on this locus. While we know that Bd is present in the known‐tolerant populations, its selective force may not be strong enough to drive the fixation of any particular MHC alleles. Other pathogens present in the system, including viruses, bacteria, or protists, may also be influencing the allele distributions. It is possible that some alleles in our dataset are important for responses to other pathogens, and only through the maintenance of these alleles are populations able to persist. High MHC polymorphism can be maintained even when overall genetic diversity is low, highlighting the importance of polymorphism at this locus (Aguilar et al. 2004).

Additionally, gene flow between populations could lead to swamping of beneficial alleles (Lenormand 2002). According to the geographic mosaic theory of coevolution, fluctuating selection pressure for Bd response across the landscape can prevent allele fixation (J. N. Thompson 1999). This fluctuating selection pressure would prevent any single MHC allele from being favored across the landscape, meaning that gene flow between populations would hinder fixation even in populations experiencing high Bd selection pressure (J. N. Thompson 1999). Boreal toads have shown patterns of high dispersal and low population genetic structure historically, indicating that there has been gene flow between populations (Trumbo et al. 2023; Lucid et al. 2021; Murphy et al. 2010). Additionally, range‐wide RAD‐seq and nuclear genome data indicate that the CO, WY, and UT populations belong to the same eastern clade while the ID and MT populations belong to a closely related middle Rocky Mountain clade, further suggesting that there has been gene flow between these populations (USFWS 2017).

Several alleles (1, 3, and 6) occurred primarily in known‐susceptible populations and the SRM “population”. Although these alleles are also present in individuals from known‐tolerant populations, the majority of their abundance comes from susceptible populations. These alleles may be increasing susceptibility to Bd, or at least do not seem to be protective against Bd. The known‐susceptible populations only had the most common alleles across our dataset, indicating a lack of rare alleles (Figure 2). Under Red Queen dynamics of host‐pathogen coevolution, we expect that rare alleles are likely more advantageous for disease response due to negative frequency‐dependent selection (Ejsmond and Radwan 2015; Phillips et al. 2018). In our dataset, rare alleles only occur in known‐tolerant populations or in populations with unknown response (Figure 2). This pattern may reflect the overall lower genetic diversity in known‐susceptible southern Rocky Mountain populations compared to other populations across the range (USFWS 2017), though we did not detect significantly lower diversity according to any of the metrics that we calculated at this locus (Table S1).

We are not able to conclusively determine whether the presence of rare alleles in known‐tolerant populations is due to selection or recent bottlenecks in known‐susceptible populations without neutral markers. However, regardless of the cause, we predict that the presence of rare alleles is beneficial for disease response (Phillips et al. 2018), and therefore populations that lack rare alleles are likely more susceptible to Bd. We also found that F_ST_, and therefore genetic differentiation, was highest when comparing known‐tolerant with known‐susceptible populations and that these values were not correlated with geographic distance between populations. This indicates that similarity among the known‐tolerant and among the known‐susceptible populations is not solely due to proximity. Additional sampling of populations, particularly those outside of the eastern clade, would help further disentangle whether these patterns are due to selection on MHC or local demographic history. To be able to directly link MHC profiles to Bd susceptibility, these additional populations would also require experimental testing for Bd response and would ideally have monitoring history and data for Bd prevalence and population declines.

### 4.2 MHC Diversity and Selection

The observed heterozygosity of known‐tolerant populations was significantly higher than that of known‐susceptible populations, indicating that MHC heterozygotes may be better protected against Bd compared to homozygotes (Figure 3). Heterozygosity of MHC has been shown to predict Bd response in other amphibian systems, where heterozygous individuals were more likely to survive infections and less likely to be infected by Bd (Savage and Zamudio 2011; Belasen et al. 2022). One of the known‐susceptible populations, Panhandle Creek‐CO, was completely homozygous despite the presence of four unique alleles in the population. The expected heterozygosity of this population is relatively high (0.72), so an observed heterozygosity of zero is surprising. We do not believe that this population is truly completely homozygous. A few of the individuals in this population (3 of 12) had relatively low read coverage (< 3000 reads) (Table S3), which may have caused us to mistakenly identify them as homozygotes using our order of magnitude cutoffs for allele calling. Individuals from other populations with low heterozygosity (Lost Lake‐CO, SRM, and 4701‐MT) had sufficiently high read coverage (> 3000 reads) with the exception of two individuals from SRM and one individual from 4701‐MT. However, even if these individuals were all truly heterozygous, the overall observed heterozygosity for these populations would still be low and therefore would not change our predictions regarding susceptibility. Observed heterozygosity in these populations is lower than expected. This may be due to subpopulation structure or homozygote advantage, but additional data are required to explore these ideas. Regardless, we expect that populations with low observed heterozygosity are at higher risk for Bd‐related declines (Savage and Zamudio 2011; Belasen et al. 2022). Future MHC studies should include within‐sample replication where possible to validate true alleles and genotyping results.

Based on the alignment of the boreal toad alleles with MHC IIβ from other species, we are able to infer the location of 13 antigen‐binding sites (Bataille et al. 2015). We found that all four of the sites under diversifying positive selection were putative antigen‐binding sites (Figure 4). This indicates that sites under selection are those that are directly interacting with antigens, potentially impacting binding efficiency to particular pathogens like Bd. Diversifying positive selection also indicates that amino acid diversity is increased at these sites, which could increase adaptive potential (Cicconardi et al. 2017). High diversity at these sites could also indicate selection for response to a broader array of pathogens or selection for rare alleles via Red Queen dynamics (Ejsmond and Radwan 2015; Phillips et al. 2018).

The 16 boreal toad alleles clustered into 2 distinct supertypes according to amino acid properties in putative antigen‐binding sites. Supertypes represent groups of alleles with functional similarity in MHC binding specificity and therefore similarity in their ability to respond to pathogens. Putative tolerance alleles were distributed in both supertypes (Figure 2), but alleles belonging to supertype two were more common in known‐tolerant populations compared to known‐susceptible populations (Figure 5). Supertypes have been shown to be predictive of disease response in other systems (Sepil et al. 2013; Schwensow et al. 2007; Smallbone et al. 2021). Several rare alleles belonging to populations with unknown response also clustered into supertype two, suggesting that these alleles function similarly to alleles primarily present in tolerant populations. Therefore, we expect that populations with higher proportions of supertype two alleles will be better protected against Bd compared to populations with primarily supertype one alleles.

### 4.3 Drivers of Intraspecific Variation to Bd Response

Intraspecific variation in Bd response among amphibian populations has been documented in several systems. For example, *Rana yavapaiensis* populations vary in Bd susceptibility across their range. This pattern seems to be driven by a combination of biotic and abiotic factors, as MHC genotypes vary across these populations while seasonality alters Bd prevalence across the landscape (Savage et al. 2011; Savage and Zamudio 2016). Populations of *Rana boylii* differ in Bd prevalence with associated variations in environmental factors like aridity and maximum temperature, as well as biotic factors including host density and the presence of introduced bullfrogs (a Bd reservoir host) (Belasen et al. 2024; Adams et al. 2017). Genetic variation in immune response loci has yet to be explored in this species. Susceptible populations of *Litoria verreauxii alpina* have distinct patterns of immune activation compared to resistant populations, indicating variation in immune response (Grogan, Cashins, et al. 2018). Populations of *Alytes obstetricans* and *Rana sylvatica* each have varying degrees of mortality following Bd exposure in a controlled laboratory setting, indicating some genetic driver of susceptibility (Tobler and Schmidt 2010; Bradley et al. 2015). Upland populations of *Physalaemus pustulosus* previously exposed to Bd show evidence of differential selection on MHC compared to lowland populations where Bd‐related decline has been less pronounced (Kosch et al. 2016).

Through all of these amphibian systems, both biotic and abiotic factors can contribute to variation in Bd response. Even if environmental factors are primarily driving responses, selection can still act on immune response loci in populations that are exposed to Bd. In the boreal toad system, there is a latitudinal gradient of response to Bd with populations in the southern Rocky Mountains more affected than populations in the north. This suggests an environmental driver of disease susceptibility (Mosher et al. 2018; Muths et al. 2003). However, Wyoming populations show evidence of higher Bd tolerance compared to Colorado populations in a controlled lab setting when individuals were raised from eggs in captivity (Hardy et al. 2025), suggesting that the environment is not the sole driver of Bd response in this system. Our results also indicate a genetic driver of Bd response, as there are differences between tolerant and susceptible populations in MHC allele distribution, observed heterozygosity, and distribution of MHC supertypes. Though the environment may play a role in boreal toad intraspecific variation, it is likely not the sole driver. Genetic differences between populations are also important for disease response and could be a helpful diagnostic tool for disease susceptibility in the face of changing environments.

### 4.4 Prediction of Bd Response in Populations With Unknown Response

For a population to be considered putatively tolerant, we expect to see high observed heterozygosity, a high proportion of supertype two alleles, and the presence of one or more putative tolerance alleles (Figure 6). The SRM “population” is very similar to the known‐susceptible populations (also located in the southern Rocky Mountain region) with low observed heterozygosity, a low proportion of supertype two alleles, and no putative tolerance alleles. Therefore, we predict that the included populations are at high risk for Bd‐related declines. In contrast, the Montana populations (4701‐MT and Jones‐MT) had a high proportion of supertype two alleles and contained putative tolerance alleles, but the 4701‐MT population had very low observed heterozygosity. Regardless, our data suggest that the 4701‐MT population has some signatures of tolerance and therefore is at lower risk of Bd‐related declines compared to the susceptible populations in our dataset. Bd is present in both Montana populations and survival of individuals in those populations is negatively impacted by Bd, though this effect size is similar to that of the known‐tolerant Blackrock‐WY population (Kain et al. 2025), supporting our hypothesis that these populations are at least somewhat tolerant to Bd.

**FIGURE 6:**
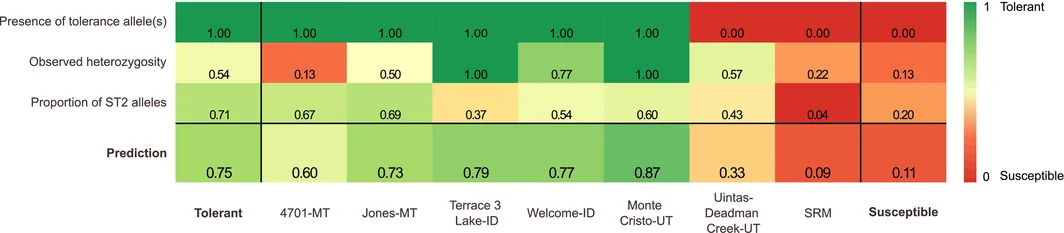
Summary of Bd response predictions for populations where Bd response has not been experimentally determined. Tolerant and susceptible predictors are the average values of the populations with known responses in this study (known‐tolerant: Blackrock‐WY and Chall Creek‐WY, known‐susceptible: Lost Lake‐CO and Panhandle Creek‐CO). Values closer to one (green) on this scale represent predicted tolerance with the presence of putative tolerance allele(s), high observed heterozygosity, and high proportion of supertype 2 alleles, while values closer to zero (red) represent predicted susceptibility with the absence of putative tolerance alleles, low observed heterozygosity, and low proportion of supertype 2 alleles. Prediction values are the average of the three predictor categories.

The Idaho populations (Terrace 3 Lake‐ID and Welcome‐ID) had high observed heterozygosity, relatively high proportions of supertype two alleles, and both contained putative tolerance alleles. Thus, we believe these populations are likely similar in tolerance to the known‐tolerant populations in Wyoming. Low sample size in the Terrace 3 Lake‐ID population (*n* = 4) may decrease our overall prediction accuracy; additional sampling in this population would increase the accuracy of heterozygosity and allele frequency estimates. There is no history of Bd monitoring in these populations, though we do know they are small and potentially facing extirpation (D. Pilliod, personal communication). Due to the genetic characteristics we identified in these populations, we suggest these declines are unlikely to be driven by Bd.

The Utah populations (Monte Cristo‐UT and Uintas Deadman Creek‐UT) had high observed heterozygosity, relatively high proportions of supertype two alleles, and one of the two (Monte Cristo‐UT) contained putative tolerance alleles. Thus, the Monte Cristo‐UT population is likely similar to the known‐tolerant populations. Because the Uintas‐Deadman Creek‐UT population does not contain any putative tolerance alleles, this population may be at higher risk for a Bd‐related decline compared to Monte Cristo‐UT, but is still likely better protected against Bd compared to susceptible populations. The lack of putative tolerance alleles in this population may also be due to low sample size (*n* = 7). Additional sampling in both Utah populations could increase the chances of allele detection and aid in prediction accuracy.

Predicting species responses to disease and other global stressors is challenging, as it can include assumptions about what “tolerant” and “susceptible” entails. We acknowledge that the survival of individuals in the known‐tolerant populations is still negatively affected by Bd (Kain et al. 2025), though to a lesser extent than known‐susceptible populations. Thus, we expect that known‐tolerant and predicted‐tolerant populations will not experience mass mortality events but may still experience slow declines. Our results do indicate a genetic driver of Bd susceptibility, but even individuals that may be genetically tolerant may succumb to Bd infections if loads are high and environmental stressors are negatively impacting the immune response. Additionally, other immune response loci are likely also contributing to Bd response, so while variation at MHC is associated with disease susceptibility in this and other systems (Matzaraki et al. 2017), it may not completely explain Bd response in all cases. Our results are predictions of future Bd response and should serve as guidance for which boreal toad populations may need more immediate conservation intervention.

### 4.5 Conservation Implications

Determining the potential genetic signatures of Bd tolerance, especially in species that vary intraspecifically in Bd response, is important in identifying individuals for reintroduction, translocation, and captive breeding efforts. These results can pave the way for the direct translation of disease research to conservation management, which is currently lacking in amphibians (Crawford‐Ash et al. 2026). While it is possible to simply select individuals from populations that persist with Bd to use in recovery actions, our ability to directly target individuals that show signatures of adaptive tolerance would likely increase the success of these efforts. Through genetic screening, individuals that are heterozygous at MHC and/or carrying beneficial alleles can be specifically chosen by managers for conservation initiatives. The introduction of putative tolerance alleles and supertype two alleles into populations that lack this diversity could increase the survival and genetic diversity of recipient populations (i.e., genetic rescue). Numerous boreal toad reintroduction efforts in Colorado and New Mexico have sourced individuals from a variety of populations in the southern Rocky Mountains, including the Lost Lake‐CO and Panhandle Creek‐CO populations. These efforts successfully established a reproducing boreal toad population in the absence of Bd, but no reintroduced population has persisted with Bd (Muths et al. 2014, 2024). Conversely, reintroduction efforts in Utah have been more successful (P. D. Thompson 2019). The source populations used in these efforts are not the same as the ones included in this study, but they are likely more similar in tolerance predictors to the Utah populations than to the Colorado populations in this study, which may have influenced reintroduction success.

As Bd continues to spread across the landscape, it is likely more advantageous to reintroduce individuals that are able to tolerate Bd rather than introducing susceptible individuals to habitats where Bd is not yet present. Reintroduction can be a time‐consuming and costly endeavor, and although screening individuals for signatures of tolerance would add an upfront time commitment and cost, the long‐term benefits of establishing new Bd tolerant populations will likely outweigh that cost. Efforts to reintroduce populations in the southern Rocky Mountains without these genetic screening tools have failed when Bd is present (Muths et al. 2014, 2024). Increasing the success rate of reintroductions could reduce the need and cost for additional reintroductions and potential failures. While Bd is certainly not the only reason that populations become extirpated or fail to establish after reintroductions, giving new populations the best possible chances of success in dealing with pathogens will surely be beneficial.

## 5 Conclusion

We found that particular MHC alleles, high observed heterozygosity, and the presence of supertype two alleles are all associated with tolerant boreal toad populations. Southern Rocky Mountain populations, including those that are known to be Bd susceptible, are similar to one another at this MHC locus and differ from known‐tolerant populations, allowing us to predict Bd response using MHC. Northern populations are variable in their respective levels of predicted tolerance, though all show signatures of increased tolerance compared to southern Rocky Mountain populations. Continued monitoring of these populations for Bd prevalence and declines, particularly for populations where we do not currently have Bd prevalence data, will be useful. Future work can experimentally test individuals from populations with unknown response to determine if our susceptibility predictions align with variation in individual tolerance. Additionally, coupling MHC genotype with the Bd infection status of each individual would allow estimation of relative risk for particular alleles.

Our results can aid conservation efforts by providing a way to predict Bd response in threatened populations of boreal toads without the need to sacrifice individuals or take tissue, instead using buccal swabs. After additional validation to ensure that the putative tolerance alleles we identified in this study are truly reducing Bd susceptibility, this approach could predict susceptibility in populations of boreal toads without the need for additional time‐consuming laboratory trials of focal populations or long‐term population mark‐recapture monitoring to determine if populations persist with Bd. Declines in populations with signatures of Bd tolerance could also suggest alternative drivers of population decline, allowing for more efficient targeting of management strategies. Our results indicate that there is an immunogenetic driver of intraspecific variation in disease susceptibility in boreal toads that can be used to target conservation efforts.

## Funding

This study was supported by funds from NSF grant DEB‐2316468.

## Conflicts of Interest

The authors declare no conflicts of interest.

## Supporting information


**Table S1:** Summary statistics of each population. Populations are categorized as T = known tolerant, S = known susceptible, or U = unknown response. The “Bd present” column indicates whether Bd has been detected in the population or if the population has not been sampled for Bd (unknown). The “Decline history” column indicates to what extent populations have undergone observed declines (“slight” = small/gradual, “dramatic” = large/pronounced). See Methods for more information. *N* = sample size, H_O_ = observed heterozygosity, H_E_ = expected heterozygosity, H_E_V = variance of expected heterozygosity, AR = raw allelic richness, ARE = allelic richness estimated with extrapolation, ARR = allelic richness estimated with rarefaction, Pi = nucleotide diversity, D = Tajima's D, DpN = uncorrected *p* value for Tajima's D assuming normal distribution, DpB = uncorrected *p* value for Tajima's D assuming beta distribution, ST1 = proportion of supertype one alleles, ST2 = proportion of supertype two alleles. See Table S2 for latitude and longitude data for the SRM “population”.
**Table S2:** Locality information for individuals included in the SRM “population”. *N* = sample size.
**Table S3:** Genotypes and read coverage per allele for all individuals included in this study. Numbers in the “Genotype” column refer to allele numbers as described in this study (e.g., 1/1 = Allele 1/Allele 1). Samples with an asterisk following the sample name are the four individuals classified as homozygous due to having a greater than 3.53× difference in the ratio of read coverage between the two alleles with the highest coverage.
**Table S4:** Pairwise comparisons of fixation index (*F*_ST_) for each population pair. Comparisons between known‐tolerant and known‐susceptible populations are bolded and darkly shaded. Comparisons between known‐tolerant and known‐tolerant or known‐susceptible and known‐susceptible populations are italicized and lightly shaded.

## Data Availability

The sequences analyzed in this study are deposited at GenBank under accession numbers PX693960–PX693975.
