# Supplementary material for: Variation in Disease Susceptibility Is Associated With Immunogenetics in a Threatened Amphibian
Source: Evol Appl. 2026 Oct 28;19(11):e70348. doi: 10.1111/eva.70348 (PMC13717072; doi:10.1111/eva.70348)
Supplement: Supplementary file 1 [file EVA-19-e70348-s001.zip › eva70348-sup-0001-Supinfo1@boreal toad MHC supplemental revised.docx]

**Supplemental Table 1** Summary statistics of each population. Populations are categorized as T = known tolerant, S = known susceptible, or U = unknown response. The “Bd present” column indicates whether Bd has been detected in the population or if the population has not been sampled for Bd (unknown). The “Decline history” column indicates to what extent populations have undergone observed declines (“slight” = small/gradual, “dramatic” = large/pronounced). See Methods for more information. N = sample size, H_O_ = observed heterozygosity, H_E_ = expected heterozygosity, H_E_V = variance of expected heterozygosity, AR = raw allelic richness, ARE = allelic richness estimated with extrapolation, ARR = allelic richness estimated with rarefaction, Pi = nucleotide diversity, D = Tajima’s D, DpN = uncorrected *p* value for Tajima’s D assuming normal distribution, DpB = uncorrected *p* value for Tajima’s D assuming beta distribution, ST1 = proportion of supertype one alleles, ST2 = proportion of supertype two alleles. See supplemental table 2 for latitude and longitude data for the SRM ‘population’.

| **Population** | **N** | **Latitude** | **Longitude** | **Bd present** | **Decline history** | **H_O_** | **H_E_** | **H_E_V** | **AR** | **ARE** | **ARR** | **Pi** | **D** | **DpN** | **DpB** | **ST1** | **ST2** |
| --- | --- | --- | --- | --- | --- | --- | --- | --- | --- | --- | --- | --- | --- | --- | --- | --- | --- |
| Blackrock  -WY (T) | 14 | 43.829806 | -110.353972 | Yes | Slight^19,39^ | 0.642 | 0.859 | 0.925 | 8 | 9.891 | 5.105 | 0.045 | 0.599 | 0.549 | 0.556 | 0.393 | 0.607 |
| Chall Creek-WY (T) | 14 | 43.021917 | -110.421722 | Yes | None^40^ | 0.428 | 0.439 | 9.531 | 6 | 7.816 | 2.851 | 0.025 | -1.131 | 0.257 | 0.271 | 0.179 | 0.821 |
| Lost Lake-CO (S) | 16 | 40.505946 | -105.598879 | Yes | Dramatic^36,41^ | 0.250 | 0.707 | 2.478 | 5 | 7.737 | 3.511 | 0.029 | -0.192 | 0.847 | 0.885 | 0.844 | 0.156 |
| Panhandle Creek-CO (S) | 12 | 40.865748 | -105.766906 | Yes | Unknown | 0 | 0.724 | 1.317 | 4 | 6.950 | 3.442 | 0.043 | 1.369 | 0.171 | 0.191 | 0.750 | 0.250 |
| SRM (U) | 27 | See supp table 2 | See supp table 2 | Yes | Dramatic^23,36,37,41^ | 0.222 | 0.554 | 11.155 | 4 | 8.897 | 2.835 | 0.014 | -1.434 | 0.151 | 0.140 | 0.963 | 0.037 |
| 4701-MT (U) | 15 | 48.479916 | -113.369994 | Yes | Slight^22,45^ | 0.133 | 0.606 | 6.320 | 5 | 9.701 | 3.281 | 0.044 | 0.914 | 0.360 | 0.379 | 0.333 | 0.667 |
| Jones-MT (U) | 16 | 46.896318 | -113.441387 | Yes | Slight^22,45^ | 0.500 | 0.620 | 7.635 | 7 | 11.203 | 3.488 | 0.050 | 1.285 | 0.198 | 0.219 | 0.312 | 0.688 |
| Terrace 3 Lake-ID (U) | 4 | 45.136112 | -114.611015 | Unknown | Unknown | 1 | 0.678 | 0.729 | 3 | 5.247 | 3 | 0.053 | 0.369 | 0.711 | 0.724 | 0.625 | 0.375 |
| Welcome  -ID (U) | 13 | 45.128524 | -114.591342 | Unknown | Unknown | 0.769 | 0.59 | 1.788 | 4 | 7.831 | 2.610 | 0.049 | 0.929 | 0.352 | 0.373 | 0.462 | 0.538 |
| Monte Cristo-UT (U) | 5 | 41.509301 | -111.509257 | Unknown | Unknown | 1 | 0.888 | 0.460 | 6 | 6.991 | 5.355 | 0.054 | 0.309 | 0.756 | 0.760 | 0.400 | 0.600 |
| Uintas-  Deadman Creek-UT (U) | 7 | 40.928874 | -110.793495 | Unknown | Unknown | 0.571 | 0.527 | 0.686 | 2 | 5.709 | 1.999 | 0.020 | 0.866 | 0.385 | 0.416 | 0.571 | 0.429 |

**Supplemental Table 2** Locality information for individuals included in the SRM ‘population’. N = sample size

| **Site Name** | **N** | **Latitude** | **Longitude** |
| --- | --- | --- | --- |
| Silverdale | 2 | 39.764113 | -105.819747 |
| Leavenworth Creek | 2 | 39.65754 | -105.778107 |
| South Cottonwood | 4 | 38.76115 | -106.345301 |
| Fourmile Creek | 2 | 38.969559 | -106.143229 |
| South Cottonwood West | 1 | 38.756409 | -106.365628 |
| Henderson Park | 1 | 39.368734 | -106.572473 |
| Triangle Pass | 1 | 38.998007 | -106.935826 |
| Cow Creek | 1 | 38.825606 | -106.443542 |
| Trout Creek | 4 | 40.957853 | -105.661407 |
| Zimmerman | 1 | 40.542793 | -105.865866 |
| Mt Dickinson | 2 | 40.936719 | -105.689207 |
| East Maroon Creek | 1 | 39.089807 | -106.917049 |
| Red Mountain | 3 | 40.373341 | -105.885391 |
| Elkhead | 1 | 40.776177 | -107.12866 |
| Ryan Park | 1 | 41.316151 | -106.485395 |

**Supplemental Table 3** Genotypes and read coverage per allele for all individuals included in this study. Numbers in the “Genotype” column refer to allele numbers as described in this study (e.g., 1/1 = Allele 1/Allele 1). Samples with an asterisk following the sample name are the four individuals classified as homozygous due to having a greater than 3.53x difference in the ratio of read coverage between the two alleles with the highest coverage.

**Supplemental Table 4** Pairwise comparisons of fixation index (F_ST_) for each population pair. Comparisons between known-tolerant and known-susceptible populations are bolded and darkly shaded. Comparisons between known-tolerant and known-tolerant or known-susceptible and known-susceptible populations are italicized and lightly shaded.

|  | **4701-MT** | **Uintas-**  **Deadman Creek-UT** | **Monte Cristo-UT** | **Blackrock**  **-WY** | **SRM** | **Chall Creek-WY** | **Jones-MT** | **Lost Lake-CO** | **Panhandle Creek-CO** | **Terrace 3 Lake-ID** | **Welcome**  **-ID** |
| --- | --- | --- | --- | --- | --- | --- | --- | --- | --- | --- | --- |
| **4701-MT** |  | 0.408 | 0.138 | 0.189 | 0.399 | 0.44 | 0.008 | 0.271 | 0.19 | 0.105 | 0.098 |
| **Uintas-**  **Deadman Creek-UT** |  |  | 0.181 | 0.278 | 0.134 | 0.516 | 0.412 | 0.142 | 0.302 | 0.426 | 0.443 |
| **Monte Cristo-UT** |  |  |  | 0.01 | 0.166 | 0.148 | 0.157 | 0.065 | 0.06 | 0.133 | 0.171 |
| **Blackrock**  **-WY** |  |  |  |  | 0.277 | *0.177* | 0.193 | 0.153 | **0.114** | 0.157 | 0.208 |
| **SRM** |  |  |  |  |  | 0.464 | 0.396 | 0.032 | 0.215 | 0.382 | 0.41 |
| **Chall Creek-WY** |  |  |  |  |  |  | 0.44 | 0.372 | **0.371** | 0.46 | 0.46 |
| **Jones-MT** |  |  |  |  |  |  |  | 0.272 | 0.194 | 0.15 | 0.12 |
| **Lost Lake-CO** |  |  |  |  |  |  |  |  | *0.094* | 0.244 | 0.295 |
| **Panhandle Creek-CO** |  |  |  |  |  |  |  |  |  | 0.175 | 0.222 |
| **Terrace 3 Lake-ID** |  |  |  |  |  |  |  |  |  |  | -0.017 |
| **Welcome**  **-ID** |  |  |  |  |  |  |  |  |  |  |  |
